# Prevalence and Factors Associated With Eating Disorder Risk Among College Students in Kerala, India: A Cross-Sectional Study

**DOI:** 10.7759/cureus.80374

**Published:** 2025-03-10

**Authors:** Swathy Krishna, Jeby Jose Olickal, P Sankara Sarma, Kavumpurathu Raman Thankappan

**Affiliations:** 1 Department of Public Health, Amrita Institute of Medical Sciences, Kochi, IND

**Keywords:** eating disorder, kerala, mental health, social media addiction, young adults

## Abstract

Introduction

Unhealthy diets are a major contributor to the global burden of disease, with eating disorders (EDs) being among the most prevalent psychological disorders affecting youth worldwide. Despite their increasing recognition, there is limited research quantifying ED risk in India. Therefore, we aimed to estimate the risk of ED in the Indian state of Kerala and to find the sociodemographic factors associated with it.

Methods

We conducted this study among 823 students (females: 60%) aged 18-22 years in randomly selected colleges in two of the 14 districts in Kerala. Data were collected using the self-administered Eating Attitudes Test-26 (EAT-26). Information on social media disorder was collected using the Bergen Social Media Addiction Scale (BSMAS). Log binomial regression analysis was done to find the factors associated with ED risk, and adjusted prevalence ratios (APR) with a 95% confidence interval (CI) were calculated.

Results

ED risk was reported by 242 students (29.4%, 95% CI: 26.3-32.6). Home-staying students are more likely to report a higher risk of ED compared to hostel or paying guest students (APR = 1.40; 95% CI: 1.08-1.80). Similarly, students belonging to high-income groups of social class I (APR = 9.60; 95% CI: 5.11-18.04), social class II (APR = 5.51; 95% CI: 2.89-10.47), and social class III (APR = 3.18; 95% CI: 1.54-6.56) were more likely to report ED risk compared to their counterparts. Additionally, students who were underweight (APR = 1.60; 95% CI: 1.24-2.05), overweight/obese (APR = 2.42; 95% CI: 1.96-3.01), and those with social media disorder (APR = 1.69; 95% CI: 1.27-2.24) had a higher likelihood of reporting ED risk.

Conclusion

Nearly one-third of college students were at risk of ED. Measures to reduce ED risk are required among students prioritizing those belonging to high-income groups, overweight/obese, underweight, and those having social media disorder.

## Introduction

Unhealthy diets are a significant contributor to the global burden of disease, with dietary risk factors accounting for 11 million deaths and 255 million disability-adjusted life years (DALYs) in 2017 [1]. Eating disorders (EDs) disrupt healthy eating patterns [2]. An ED is defined as any disorder characterized primarily by a pathological disturbance of attitudes and behaviors related to food, including anorexia nervosa (AN), bulimia nervosa (BN), and binge-eating disorder (BED) [3]. EDs are among the most common psychological disorders affecting the youth worldwide [4]. According to the Global Burden of Diseases Study, an estimated 13.6 million people, equivalent to 176.2 per 100,000, had AN or BN in 2019. Additionally, a study by Santomauro et al. estimated 41.9 million prevalent cases of BED and other specified feeding or eating disorders (OSFED) globally in 2019, equivalent to 541.1 per 100,000 people [4].

Sociodemographic factors, such as age, gender, and socioeconomic status significantly influence the risk of developing EDs. For example, females and adolescents are particularly vulnerable [5,6]. Similarly, cultural and racial pressures, combined with low self-esteem and high body image preoccupation, further exacerbate susceptibility [7]. Social media use has also been linked to an increased risk of EDs, as platforms such as Instagram and TikTok often promote unrealistic body ideals, leading to body dissatisfaction and disordered eating behaviors [8,9]. The frequent exposure to appearance-focused content and engagement in social comparison intensify these risks, particularly among adolescents and young adults. Additionally, social media platforms expose children and adolescents to various forms of food marketing, most of which promote unhealthy food choices [10].

Compared to other Indian states, Kerala is in a more advanced stage of epidemiological transition [11]. This is evident from the finding that the proportion of total disease burden from non-communicable diseases (NCDs) in Kerala is 74.6% in the age group of 15-69 years, which is higher than in other Indian states. Research on EDs in India is limited, with a few studies examining associated risk factors and the influence of social media. For instance, a study conducted in Mysore in the neighboring state of Karnataka found that 26.06% of students exhibited abnormal eating attitudes, indicating a significant prevalence of EDs among university students [12]. However, this study did not extensively explore the socio-demographic factors contributing to these disorders. There have been no studies conducted in Kerala on EDs, leaving a gap in understanding their prevalence and associated factors in this unique socio-cultural context. Therefore, this study aimed to estimate the risk of EDs among college students aged 18-22 years in Kerala and to find the sociodemographic factors associated with the risk of EDs.

## Materials and methods

Study design

This study was a college-based cross-sectional study.

Study setting

Kerala is the 21st largest Indian state, with 14 districts and a population of 33 million as per census 2011. This study was conducted in selected colleges from two districts: Kollam in the South and Kozhikode in the north of Kerala. Government colleges, colleges run by private management with financial support from the government (aided colleges), and private arts and science and engineering colleges with no financial aid from the government were included. No routine health campaigns have been conducted in these selected colleges to promote healthy dietary habits in students in the post-COVID period.

Study population

Students aged 18-22 years pursuing arts and science or engineering courses in Kollam and Kozhikode districts are included. 

Study duration

The study duration was eight months from November 2023 to May 2024. The data collection period was from February 15, 2024, to April 15, 2024.

Sample size

The sample size was estimated assuming the proportion of college students at risk of EDs as 26.06% [12], with 5% absolute precision and a design effect of two; the calculated sample size for the study was 592. The sample size was calculated using Open Epi Version 3.01 (version 7.1.5; Centers for Disease Control and Prevention, Atlanta, GA). After considering a non-response rate of 20%, the final sample size for the study was 740. However, we have included all the students from the selected clusters (class divisions), and the final sample size used for the analysis was 823.

Sampling technique 

**Figure 1:**
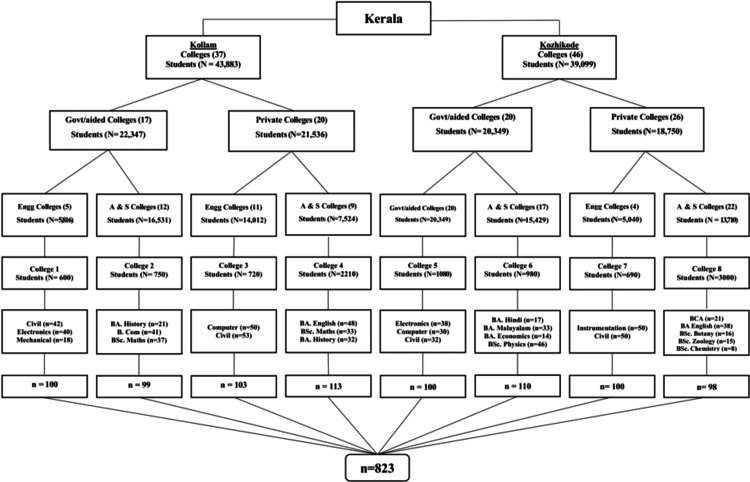
Flowchart depicting the selection of study participants

Among the 14 districts in Kerala, two districts (Kollam and Kozhikode) were randomly selected from the seven Southern and seven Northern districts of Kerala. In Kollam, 22,347 students were enrolled in government colleges, including aided colleges, and 21,536 students in private colleges. In Kozhikode, 20,349 students were enrolled in government colleges, including aided colleges, and 18,750 students in private colleges. From each district, two arts and science and two engineering colleges, both of government/aided and private sector, were selected by a simple random sampling (lottery method). The class divisions were selected from the colleges chosen using a computer-generated simple random sampling method. All the students present in the classroom during the data collection were included. The detailed sample selection technique is given in Figure 1. 

Study tools

A self-administered structured and validated questionnaire was used. The ED risk was assessed by the Eating Attitudes Test-26 (EAT-26) questionnaire. The questionnaire was originally developed and validated by Garner et al. [13]. Permission to use EAT-26 was obtained from Dr. David M. Garner, the original author. The full questionnaire is provided in the appendix. This is a 26-item scale, with each item answered on a six-point Likert scale ranging from “never” to “always.” The most symptomatic response is assigned a score of three, the next most symptomatic response a score of two, and the least symptomatic a score of one. The remaining three choices are assigned a score of zero. Total scores are derived as a sum of the composite items, ranging from zero to 78, with the 26th question reverse scored. Scores greater than 20 indicated abnormal eating attitudes and behaviors and were considered to have a risk of ED.

To assess social media disorder (SMD), a validated six-item scale, the Bergen Social Media Addiction Scale (BSMAS), was used. The BSMAS is reproduced from Andreassen et al. [14] with proper attribution. The scale is freely available for academic research purposes. No additional permissions were required for its use in this study. The BSMAS is freely accessible, and it has been used in studies conducted previously in India. The scale uses a five-point Likert scale, ranging from one (very rarely) to five (very often). A score of 24 and above was used as a clinical cut-off point for SMD. 

Procedure of data collection

Permission from the higher officials of each college was sought before data collection. Questionnaires were distributed to the students of the selected class divisions, and a brief explanation of the purpose of the study was given. It took about 20-30 minutes to complete the data collection in each class division.

Independent and outcome variables

The independent variables were age, gender (male, female, transgender), marital status (married or unmarried), social class (I/higher income group, II, III, IV, V/lowest income group based on revised BG Prasad's classification 2024) [15], religion (Hindu, Christian, Muslim, or others), course pursuing (engineering and arts & science courses such as B Com, BA, and other BSc and MSc courses), self-reported weight and height, and SMD. The outcome variable was the risk of ED (Yes/No).

Ethical considerations

A written informed consent was obtained before distributing the questionnaires. Participants were informed that participation in the study was voluntary and that their responses would be treated with the strictest confidence. No sensitive personal data were collected from the participants.

Statistical analysis

The collected data were entered into EpiData Version 4, and the statistical analyses were performed using STATA Version 14 (StataCorp LLC, College Station, TX). The categorical variables, such as gender, course pursued in college, type of college, sibling status, place of stay, religion, marital status, and socioeconomic class, were summarized as frequencies and percentages. The continuous variables such as age, monthly household income, duration of daily social media use, and SMD were summarized as mean (SD). The prevalence of ED risk was calculated with 95% confidence intervals (CI). The factors associated with ED risk were assessed using the chi-squared test. Variables with a p-value of less than 0.2 in the bivariate analysis were included in the multivariable analysis using a log-binomial model to estimate the adjusted PR (APR) with 95% CI. A p-value less than 0.05 was considered statistically significant.

## Results

Of the 823 participants, nearly two-fifths (n=327, 39.7%) were males and the mean (SD) age was 19.8 (1.3) years. Table 1 depicts the sociodemographic characteristics of the participants. An equal number of students from government/aided and private colleges have participated. Of the total sample, 415 (50.4%) were from Kollam districts, and 805 (97.8%) students were unmarried. About four-fifths (n=607, 73.5%) of the students were coming from home, 732 (89%) students had siblings, and about 125 (15%) were overweight or obese.

**Table 1:** Distribution of sociodemographic characteristics (n=823) *Social class: Based on modified BG Prasad’s scale for 2024 (calculated using monthly household income/total number of family members). †Based on self-reported weight and height. §Atheist, n=3

Variables	n	%
Age in years		
18	148	18.0
19	204	24.8
20	225	27.3
21	114	13.9
22	132	16.0
Gender		
Male	327	39.7
Female	496	60.3
College		
Government	310	37.7
Aided	99	12.0
Private	414	50.3
Course		
B.Tech	403	49.0
Arts and Science	420	51.0
District		
Kozhikode	408	49.6
Kollam	415	50.4
Social-class*		
Class 1 (³₹9,098)	251	30.5
Class 2 (₹4,549-9,097)	235	28.5
Class 3 (₹2,729-4,548)	142	17.3
Class 4 (₹1,364-2,728)	147	17.9
Class 5	48	5.8
Place of stay		
Home	607	73.8
Hostel	158	19.2
Paying guest	58	7.0
Marital status		
Married	18	2.2
Unmarried	805	97.8
Religion		
Hindu	441	53.6
Muslim	235	28.5
Christian	144	17.5
Others^§^	3	0.4
Sibling status		
Yes	732	88.9
No	91	11.1
Body mass index (BMI) ^†^		
Underweight (<18.5 kg/m^2^)	233	28.3
Normal (18.5-24.9 kg/m^2^)	465	56.5
Overweight (25-29.9 kg/m^2^)	98	11.9
Obese (30 kg/m^2 ^and above)	27	3.3

Table 2 shows the participants' eating behaviors related to social media use. Three-fourths (n=620, 75.3%) were interested in watching food-related social media posts. More than half of the participants used social media to follow accounts that promote restaurants (n=471, 57.2%) and body fitness (n=473, 57.5%), and 352 (42.8%) reported an increase in fast-food consumption after they started using social media.

**Table 2:** Self-reported perceptions of social media influence on eating habits and fitness behavior (n=823) *Multiple answers are possible.

Variables	n	%
Follow social media accounts that promote restaurants	471	57.2
Social media posts influence what you choose to eat in a day	290	35.2
Interested in food-related posts shared on social media and consume that food	620	75.3
Fast food consumption increased after starting social media use	352	42.8
Use social media for body fitness	473	57.5

Item-wise response to the EAT-26 questionnaire is provided in the appendix. A significant proportion of respondents reported never experiencing behaviors associated with disordered eating, particularly vomiting after meals (521, 63.30%) and feeling that food controls their lives (285, 34.63%). However, a notable percentage often or sometimes exhibited a preoccupation with food (157, 19.08%; 228, 27.70%, respectively), calorie awareness (116, 14.09%; 160, 19.44%), and dieting behaviors (153, 18.59%; 162, 19.68%). Some respondents also expressed concern about body weight, with 328 (39.85%) never being occupied with a desire to be thinner, while 129 (15.67%) often and 173 (21.02%) sometimes worried about body fat. The highest always response was for enjoying trying new rich foods (146, 17.74%).

The prevalence of the risk of ED among young adults was 29.4% (n=242, 95% CI: 26.3-32.6). Table 3 presents the bivariate analysis of sociodemographic factors and social media usage associated with the risk of EDs. Social class, place of stay, BMI, and SMD were significantly associated with ED risk.

**Table 3:** Sociodemographic and social media usage factors associated with the risk of ED: Bivariate analysis results (n=823) ED=eating disorder

Variables	n	ED Risk	No ED Risk	χ2 value	P-value
		n (%)	n (%)		
ED	823	242 (29.4)	581 (70.6)		
Age in years					
18	148	44 (29.7)	104 (70.3)	2.12	0.713
19	204	62 (30.4)	142 (69.6)		
20	225	70 (31.1)	155 (68.9)		
21	114	34 (29.8)	80 (70.2)		
22	132	32 (24.2)	100 (75.8)		
Gender					
Female	496	149 (30.0)	347 (70.0)	0.248	0.622
Male	327	93 (28.4)	234 (71.6)		
Course					
B. Tech	403	126 (31.3)	277 (68.7)	1.32	0.251
Arts and science	420	116 (27.6)	304 (72.4)		
District					
Kollam	415	131 (31.6)	284 (68.4)	1.88	0.170
Kozhikode	408	111 (27.2)	297 (72.8)		
Social-class					
Class 1	251	141 (56.2)	110 (43.8)	155.26	<0.001
Class 2	235	69 (29.4)	166 (70.6)		
Class 3	142	22 (15.5)	120 (84.5)		
Class 4 & 5	195	10 (5.1)	185 (94.9)		
Place of stay					
Home	607	192 (31.6)	415 (68.4)	5.52	0.019
Hostel/ Paying guest	216	50 (23.1)	166 (76.8)		
Marital status					
Married	18	9 (50.0)	9 (50.0)	3.76	0.052
Unmarried	805	233 (28.9)	572 (71.1)		
Religion					
Muslim	235	74 (31.5)	161 (68.5)	1.08	0.582
Christian/others*	147	45 (30.6)	102 (69.4)		
Hindu	441	123 (27.9)	318 (72.1)		
Sibling status					
No	91	32 (35.2)	59 (64.8)	0.163	0.200
Yes	732	210 (28.7)	522 (71.3)		
Body Mass Index (BMI)					
Underweight	233	68 (29.2)	165 (70.8)	108.65	<0.001
Overweight/ Obese	125	84 (67.2)	41 (32.8)		
Normal	465	90 (19.4)	375 (80.7)		
Social Media Disorder (SMD)					
SMD	38	21 (55.3)	17 (44.7)	12.83	<0.001
Normal	785	221 (28.2)	564 (71.9)		

Table 4 presents the log-binomial regression analysis of sociodemographic factors and social media usage associated with the risk of EDs. Compared to respondents staying in hostels or as paying guests, those staying at home were 1.40 times more likely to report an ED risk (APR = 1.40; 95% CI: 1.08-1.80; p = 0.010). Similarly, those in social class I (APR = 9.60; 95% CI: 5.11-18.04; p < 0.001), class II (APR = 5.51; 95% CI: 2.89-10.47; p < 0.001), and class III (APR = 3.18; 95% CI: 1.54-6.56; p = 0.002) were at significantly higher risk. Underweight individuals (APR = 1.60; 95% CI: 1.24-2.05; p < 0.001) and those who were overweight or obese (APR = 2.42; 95% CI: 1.96-3.01; p < 0.001) also showed an elevated risk. Additionally, individuals with social media disorder were 1.69 times more likely to develop an ED risk (APR = 1.69; 95% CI: 1.27-2.24; p < 0.001).

**Table 4:** Sociodemographic and social media usage factors associated with the risk of ED: Log binomial regression analysis results (n=823) ED=eating disorder, APR=adjusted prevalence ratio *Variables that had a p-value < 0.2 in the bivariate analysis are used in the regression model. We assessed how social class and social media disorder interact to influence ED risk. The interaction term (social class × social media disorder) in the model was not significant; therefore, the final model was presented without it.

Variables	APR (95% CI)	P-value	
			District			
Kollam	1.05 (0.87-1.29)	0.570	
Kozhikode	1	-	
Social class			
Class 1	9.54 (5.11-17.84)	<0.001	
Class 2	5.51 (2.90-10.47)	<0.001	
Class 3	3.17 (1.54-6.53)	0.002	
Class 4 & 5	-	-	
Place of stay			
Home	1.40 (1.08-1.80)	0.001	
Hostel/Paying guest	-	-	
Marital status			
Married	1.18 (0.82-1.70)	0.390	
Unmarried	-	-	
Sibling status			
No	1.09 (0.84-1.42)	0.518	
Yes	-	-	
Body Mass Index (BMI)			
Underweight	1.61 (1.25-2.06)	<0.001	
Overweight/Obese	2.43 (1.96-3.02)	<0.001	
Normal	-	-	
Social Media Disorder (SMD)			
SMD	1.67 (1.26-2.22)	<0.001	
Normal	-	-	

## Discussion

This study investigated the prevalence of the risk of ED and the socio-demographic factors and social media use associated with it among college students aged 18-22 years in Kerala. The results show that the prevalence of the risk of ED among young adults was 29.4%. Similar to our study, research by Nivedita et al. reported the risk of ED among medical students in south India as 26.06% [12]. The sociocultural differences between Kerala and Mysore may contribute to variations in ED prevalence, influenced by factors such as dietary practices, beauty standards, and media exposure. A high level of packaged food consumption was reported in Kerala among adults aged 18-30 years in a study by Haseena et al. [16]. International studies have reported varying ED prevalence rates, with higher rates observed in Western countries where sociocultural emphasis on thinness and body image is more pronounced, while lower prevalence is noted in some Asian countries, possibly due to cultural differences in food attitudes and body perception [17,18]. Although it has been known for some time that EDs pose several health risks, Arcelus et al. in their meta-analysis in 2011 found high mortality rates in patients with EDs. This elevated risk of death was most pronounced among individuals with AN, as evidenced by a weighted annual mortality rate of five per 1,000 person-years [17]. An examination of obesity and EDs by Marcus presented that individuals exhibiting symptoms of disordered eating are especially prevalent among those with higher body weight, regardless of racial/ethnic background, socioeconomic status (SES), age, or severity of obesity [18].

Data from the GBD study suggest that an unhealthy diet is the most important behavioral risk factor for NCDs, accounting for 11 million deaths globally, and India had a significantly higher diet-related death rate compared to high-income countries [1]. Age-standardized mortality rates attributable to a diet low in fiber in 2019 were highest among Southeast Asia regions [19]. Kerala has the highest prevalence of most NCDs among the Indian states; for example, the state has a 25.5% diabetes prevalence, the highest among the major Indian states [20]. The high prevalence of risk of EDs among young adults highlights the critical need for integrated public health initiatives that address the emerging issue of EDs, particularly given that EDs can contribute to the development of NCDs.

The current study found that underweight and overweight individuals have a high risk of ED. These findings must be interpreted with caution; traditionally, EDs were viewed as conditions primarily affecting individuals with low body weight. However, a growing body of evidence now refutes this misconception. The most prevalent EDs, including BED, OSFED, and BN, can manifest across a spectrum of body weights. In addition, EDs represent a significant and growing public health concern, with a particularly concerning rise in prevalence among individuals of higher body weight [21]. For those in social class I, the risk of ED was much higher. This finding of our study is contrary to prior research conducted by Burke et al., which identified that participants from lower SES exhibited a 1.27-fold greater likelihood of screening positive for an ED compared to their higher SES counterparts [22]. The findings from studies exploring socioeconomic factors concerned with EDs vary widely; for instance, no association was found between ED features and in a study by Mulders et al. [23]. Additionally, Nagata et al. stated that lower household income was associated with both BED and binge-eating behavior in household incomes [24]. Most studies reported a null effect of socioeconomic status; however, some studies reported an association with lower socioeconomic status [5].

The results of the present study also demonstrated that the participants with SMD had a high risk of ED. A systematic review by Purba et al. previously reported that young people exposed to health-risk behavior content on social media, particularly marketer-generated content, exhibited a significant increase in unhealthy food consumption compared to adolescents with no such exposure [25]. Additional evidence comes from recent studies showing that young people acquire access to social media platforms at a significantly earlier age compared with the older generations [26]. This makes them more susceptible to social media addiction as they feel more comfortable using social media for communication [27]. Evidence of a possible association between social networking site (SNS) usage and the risk of ED comes from a meta-analysis conducted by Zhang et al. [28]. Notably, Instagram, as a visually driven social media platform, might induce body dissatisfaction and disordered eating mediated by appearance comparison processing through celebrity worship, according to a study by Brown et al. [29]. Additionally, a systematic review done by Holland et al suggested that a limited number of studies showed that social comparison based on appearance mediates the relationship between SNS use and body image and eating concerns [8]. In contrast, a review of reviews conducted by Stiglic et al. reported no or insufficient evidence for an association of screen time with EDs [30].

Similarly, more than half of the participants who used social media in the present study were following accounts that promote restaurants (57.2%) and body fitness (57.5%), and 42.8% reported an increase in fast-food consumption after they started using social media. The findings of this investigation were in line with a meta-analysis done by Zhang et al. [28], which revealed a positive association between excessive use of SNSs and the risk of developing disordered eating behaviors. Furthermore, their analysis indicated a stronger correlation between SNS usage and disordered eating behaviors among university students compared to other populations [28]. Filippone et al.previously reported a correlational relationship between social media time exposure and cognitive impulsivity and demonstrated a cross-sectional relationship between social media time exposure and food craving through an increase in cognitive impulsivity levels [23]. 

To the best of our knowledge, this is the first study from India looking at the factors associated with ED. However, this research has a few limitations. We included colleges from only two districts (one from the northern districts and another from the southern districts); studies among a larger population may provide better generalizability. Our study did not specifically examine different social media behaviors, such as engagement with body image-focused content. While our findings indicate a significant association between social media disorder and ED risk, the cross-sectional design of this study prevents us from inferring causality. Additionally, the use of self-reported height and weight for BMI calculation may introduce reporting bias, although college students are generally aware of their weight and height. Moreover, response bias and social desirability bias cannot be ruled out, as participants may have underreported or overreported their eating behaviors. 

## Conclusions

Nearly one-third of the college students were at risk of ED. Students from high-income backgrounds; those who were overweight, obese, or underweight; and those experiencing social media disorder were significantly more likely to report ED risk. The findings emphasize the urgent need for targeted interventions, particularly in high-risk groups, to promote healthy eating behaviors and mental well-being. Given the role of social media in influencing dietary habits, awareness programs addressing the impact of digital exposure on eating attitudes should be integrated into students’ health initiatives. Additionally, routine mental health screenings and accessible support services within educational institutions can help in early identification and management of ED risk. Addressing these factors through a multi-pronged approach involving students, educators, parent teacher associations, and healthcare professionals is crucial in mitigating the growing burden of EDs among young adults.

## Appendices

**Table 5:** Questionnaire: Eating Attitudes Test-26 (EAT-26) Eating Attitudes Test-26 (EAT-26) Questionnaire Source: Garner et al. [13] Permission statement: Permission to use the EAT-26 questionnaire was obtained from Dr. David M. Garner, the original author. Copyright: The EAT-26 is copyrighted by Dr. David M. Garner and Eating Attitudes, LLC. Any reproduction or use beyond this study requires additional permission. BSMAS Scale: The scale is freely available for academic research purposes. No additional permissions were required for its use in this study.

S. No.	Question			Response Options			
1	Name			________			
2	Age (in years)			________			
3	Gender			☐ Male ☐ Female ☐ Transgender			
4	Type of college			☐ Government ☐ Aided ☐ Private			
5	Course pursuing			________			
6	Place of residence			________			
7	Place of stay			☐ Home ☐ Hostel ☐ Paying guest			
8	Marital status			☐ Married ☐ Unmarried			
9	Religion			☐ Hindu ☐ Muslim ☐ Christian ☐ Other (Specify: ________)			
10	Number of household members			________			
11	Monthly household income			________			
12	Sibling status			☐ Yes ☐ No			
13	Height (in cms)			________			
14	Weight (in kg)			________			
15	Do you follow any social media accounts that promote restaurants or eateries?			☐ Yes ☐ No			
16	Do social media posts by influencers impact what you choose to eat in a day?			☐ Yes ☐ No			
17	Are you interested in food-related posts shared by influencers on social media and consuming that food?			☐ Yes ☐ No			
18	Do you think your fast-food consumption increased after you started using social media?			☐ Yes ☐ No			
19	Do you follow any accounts on social media that focus on fitness, dieting, or body image?			☐ Yes ☐ No			
20	You spend a lot of time thinking about social media or planning how to use it.			☐ Very rarely ☐ Rarely ☐ Sometimes ☐Often ☐ Very often			
21	You feel an urge to use social media more and more.			☐ Very rarely ☐ Rarely ☐ Sometimes ☐Often ☐ Very often			
22	You use social media in order to forget about personal problems.			☐ Very rarely ☐ Rarely ☐ Sometimes ☐Often ☐ Very often			
23	You have tried to cut down on the use of social media without success.			☐ Very rarely ☐ Rarely ☐ Sometimes ☐Often ☐ Very often			
24	You become restless or troubled if you are prohibited from using social media.			☐ Very rarely ☐ Rarely ☐ Sometimes ☐Often ☐ Very often			
25	You use social media so much that it has had a negative impact on your job/studies.			☐ Very rarely ☐ Rarely ☐ Sometimes ☐Often ☐ Very often			
EAT-26 Questionnaire							
	Question	Always	Usually	Often	Sometimes	Rarely	Never
26	I am terrified about being overweight.	☐	☐	☐	☐	☐	☐
27	I avoid eating when I am hungry.	☐	☐	☐	☐	☐	☐
28	I find myself preoccupied with food.	☐	☐	☐	☐	☐	☐
29	I have gone on eating binges where I feel that I may not be able to stop.	☐	☐	☐	☐	☐	☐
30	I cut my food into small pieces.	☐	☐	☐	☐	☐	☐
31	I am aware of the calorie content of foods that I eat.	☐	☐	☐	☐	☐	☐
32	I particularly avoid food with a high carbohydrate content (i.e., bread, rice, potatoes, etc.).	☐	☐	☐	☐	☐	☐
33	I feel that others would prefer if I ate more.	☐	☐	☐	☐	☐	☐
34	I vomit after I have eaten.	☐	☐	☐	☐	☐	☐
35	I feel extremely guilty after eating.	☐	☐	☐	☐	☐	☐
36	I am occupied with a desire to be thinner.	☐	☐	☐	☐	☐	☐
37	I think about burning up calories when I exercise.	☐	☐	☐	☐	☐	☐
38	Other people think that I am too thin.	☐	☐	☐	☐	☐	☐
39	I am preoccupied with the thought of having fat on my body.	☐	☐	☐	☐	☐	☐
40	I take longer than others to eat my meals.	☐	☐	☐	☐	☐	☐
41	I avoid foods with sugar in them.	☐	☐	☐	☐	☐	☐
42	I eat diet foods.	☐	☐	☐	☐	☐	☐
43	I feel that food controls my life.	☐	☐	☐	☐	☐	☐
44	I display self-control around food.	☐	☐	☐	☐	☐	☐
45	I feel that others pressure me to eat.	☐	☐	☐	☐	☐	☐
46	I give too much time and thought to food.	☐	☐	☐	☐	☐	☐
47	I feel uncomfortable after eating sweets.	☐	☐	☐	☐	☐	☐
48	I engage in dieting behavior.	☐	☐	☐	☐	☐	☐
49	I like my stomach to be empty.	☐	☐	☐	☐	☐	☐
50	I have the impulse to vomit after meals.	☐	☐	☐	☐	☐	☐
51	I enjoy trying new rich foods.	☐	☐	☐	☐	☐	☐

**Table 6:** Response to the Eating Attitude Test-26 (EAT-26) questionnaire by the students (n=823)

S. No.	Question	Always n (%)	Usually n (%)	Often n (%)	Sometimes n (%)	Rarely n (%)	Never n (%)
1	I am terrified about being overweight.	110 (13.37)	87 (10.57)	74 (8.99)	99 (12.03)	79 (9.60)	374 (45.44)
2	I avoid eating when I am hungry.	29 (3.52)	48 (5.83)	85 (10.33)	170 (20.66)	148 (17.98)	343 (41.68)
3	I find myself preoccupied with food.	37 (4.50)	83 (10.09)	157 (19.08)	228 (27.70)	148 (17.98)	170 (20.66)
4	I have gone on eating binges where I feel that I may not be able to stop.	23 (2.79)	63 (7.65)	174 (21.14)	170 (20.66)	119 (14.46)	274 (33.29)
5	I cut my food into small pieces.	76 (9.23)	82 (9.96)	168 (20.41)	215 (26.12)	137 (16.65)	145 (17.62)
6	I am aware of the calorie content of foods that I eat.	87 (10.57)	89 (10.81)	116 (14.09)	160 (19.44)	113 (13.73)	258 (31.35)
7	I particularly avoid food with a high carbohydrate content (i.e., bread, rice, potatoes, etc.)	39 (4.74)	81 (9.84)	124 (15.07)	151 (18.35)	117 (14.22)	311 (37.79)
8	I feel that others would prefer if I ate more.	47 (5.71)	70 (8.51)	108 (13.12)	143 (17.38)	133 (16.16)	322 (39.13)
9	I vomit after I have eaten.	8 (0.97)	16 (1.94)	21 (2.55)	86 (10.45)	128 (15.55)	564 (68.53)
10	I feel extremely guilty after eating.	32 (3.89)	28 (3.40)	73 (8.87)	101 (12.27)	124 (15.07)	465 (56.50)
11	I am occupied with a desire to be thinner.	55 (6.68)	79 (9.60)	108 (13.12)	150 (18.23)	103 (12.52)	328 (39.85)
12	I think about burning up calories when I exercise.	118 (14.34)	123 (14.95)	150 (18.23)	148 (17.98)	81 (9.84)	203 (24.67)
13	Other people think that I am too thin.	142 (17.25)	73 (8.87)	78 (9.48)	124 (15.07)	114 (13.85)	292 (35.48)
14	I am preoccupied with the thought of having fat on my body.	81 (9.84)	86 (10.45)	129 (15.67)	173 (21.02)	105 (12.76)	249 (30.26)
15	I take longer than others to eat my meals.	90 (10.94)	62 (7.53)	107 (13.00)	173 (21.02)	157 (19.08)	234 (28.43)
16	I avoid foods with sugar in them.	41 (4.98)	72 (8.75)	112 (13.61)	168 (20.41)	125 (15.19)	305 (37.06)
17	I eat diet foods.	32 (3.89)	93 (11.30)	143 (17.38)	177 (21.51)	149 (18.10)	229 (27.83)
18	I feel that food controls my life.	50 (6.08)	66 (8.02)	101 (12.27)	201 (24.42)	120 (14.58)	285 (34.63)
19	I display self-control around food.	81 (9.84)	111 (13.49)	149 (18.10)	203 (24.67)	120 (14.58)	159 (19.32)
20	I feel that others pressure me to eat.	88 (10.69)	102 (12.39)	125 (15.19)	159 (19.32)	121 (14.70)	228 (27.70)
21	I give too much time and thought to food.	53 (6.44)	74 (8.99)	123 (14.95)	184 (22.36)	167 (20.29)	222 (26.97)
22	I feel uncomfortable after eating sweets.	46 (5.59)	82 (9.96)	106 (12.88)	151 (18.35)	142 (17.25)	296 (35.97)
23	I engage in dieting behavior.	32 (3.89)	88 (10.69)	153 (18.59)	162 (19.68)	131 (15.92)	257 (31.23)
24	I like my stomach to be empty.	40 (4.86)	95 (11.54)	157 (19.08)	125 (15.19)	132 (16.04)	274 (33.29)
25	I have the impulse to vomit after meals.	5 (0.61)	13 (1.58)	34 (4.13)	101 (12.27)	149 (18.10)	521 (63.30)
26	I enjoy trying new rich foods.	146 (17.74)	123 (14.95)	112 (13.61)	222 (26.97)	131 (15.92)	89 (10.81)
